# TB treatment support strategies for children, adolescents, and young adults in a low-incidence setting

**DOI:** 10.5588/ijtldopen.24.0571

**Published:** 2025-09-10

**Authors:** A.R. Verhage, A.C. Hesseling, G.H. Koppelman, H.A.M. Kerstjens, O.W. Akkerman

**Affiliations:** ^1^ Department of Paediatric Infectious Diseases and Immunology, Beatrix Children’s Hospital, University of Groningen, University Medical Center Groningen, Groningen, The Netherlands;; ^2^ Desmond Tutu TB Centre, Department of Paediatrics and Child Health, Stellenbosch University, Cape Town, South Africa;; ^3^ Department of Paediatric Pulmonology and Paediatric Allergology, Beatrix Children’s Hospital, University of Groningen, University Medical Center Groningen, Groningen, The Netherlands;; ^4^ Groningen Research Institute for Asthma and COPD (GRIAC), University of Groningen, University Medical Center Groningen, Groningen, The Netherlands;; ^5^ Department of Pulmonary Diseases and Tuberculosis, University of Groningen, University Medical Center Groningen, Groningen, The Netherlands;; ^6^ TB Center Beatrixoord, University of Groningen, University Medical Center Groningen, Groningen, The Netherlands.

**Keywords:** tuberculosis, monitoring, support, treatment adherence

## Abstract

**BACKGROUND:**

Globally, TB programmes should pay attention to the treatment support needs of children and adolescents (0–24 years) given the high disease burden and specific care requirements. We examine how health care workers in a low-incidence setting monitor and support TB treatment and TB preventive treatment (TPT) in this population.

**METHODS:**

A quantitative web-based cross-sectional survey was conducted from 1 December 2023 to 31 January 2024 among Dutch health care workers routinely caring for persons (0–24 years) in community- and hospital-based TB services.

**RESULTS:**

Ninety-three health care workers participated. The most common strategies to monitor TB treatment and TPT were 1) verbal questioning on adherence (100% vs. 99%) and 2) evaluating clinical response to TB treatment (91%). Additional strategies were always used for TB treatment, with a pill organiser being the preferred method, while 50% seldom used extra strategies for TPT. Digital support technologies were rarely used for TB treatment and TPT by 78% and 90% of respondents, respectively.

**CONCLUSION:**

Dutch health care workers relied on traditional methods to support TB treatment adherence with limited use of digital technologies and greater focus on disease than infection. Further research is needed to assess whether these strategies meet young people’s needs in TB care and improve outcomes.

In 2022, 1.8 million children and adolescents and young adults (AYAs, aged 10–24 years) developed TB disease (TBD) according to the World Health Organization (WHO).^1^ The lifetime risk of developing TBD after acquiring TB infection (TBI) is estimated to be 5%–10%, but this is higher in children under 5 years and AYAs.^2–4^

A recent systematic review found TB preventive therapy (TPT) to be effective in 91% of children and adolescents with TBI, though overall effectiveness varies in the literature.^2,3^ TPT is seen as a key strategy for eliminating TB.^2,5^

Adherence to TB treatment and TPT remains a challenge in children and AYAs. Factors like pill burden, lack of paediatric formulations, palatability, and duration of treatment are important factors for non-adherence.^6^ AYAs encounter a critical period of biological growth and social role changes with greater autonomy, extensive socialisation, increased risk-taking behaviour, and a focus on short-term gains over long-term health outcomes.^7–9^

Poor adherence is described in several studies, mostly in low- and middle-income countries.^8,9^ A study in a low-incidence setting showed a completion rate of 74.7% for TPT and TB treatment in children (aged 0–18 years). Non-adherence was associated with being adolescent, being foreign born, and having language barriers.^10^ A nationwide analysis performed in children aged 0–18 years with TB in the Netherlands from 1993 to 2018 found a 92.8% TB treatment completion rate among adolescents with TBD (15–18 years), with 6.5% lost to follow-up (LTFU) in this group. While the overall mortality rate was low (22 patients, 0.7%), nearly half of the deaths occurred in this age group (10 patients).^11^ TPT completion rates were not routinely reported in the Netherlands.

There is a growing recognition that TB control programmes worldwide should prioritise vulnerable populations of children and AYAs to improve adherence and outcomes.^9^

Strategies to enhance medication adherence in children and AYAs with TB should address age-related factors, health literacy, social determinants, psychological dynamics, stigma, health care system factors, and the role of caregivers.^9^

Various strategies to monitor and support TB treatment adherence are described in literature, including directly observed therapy (DOT), pill organisers, health education, and socio-economic and psycho-emotional support.^12^

Digital technologies (DTs), such as video-observed therapy (VOT), WhatsApp/SMS reminders, and electronic pill bottles, offer promising, flexible, and efficient alternatives.^12–14^

There is limited knowledge about the strategies used to monitor and support treatment adherence in children and AYAs with TBD and TBI in low-incidence settings like the Netherlands, where increased migration and planned decentralised TB services will further burden local health services in providing TB care.

The main objective of the study was to assess the strategies used by health care workers (HCWs) in routine care to monitor and support adherence to TPT and TB treatment in children and AYAs in the Netherlands. We explored whether differences exist in the strategies employed for TPT versus TB treatment and which categories of HCWs are predominantly involved in monitoring and supporting adherence. Additionally, we investigated which patient factors HCWs believe are associated with adherence. The study also evaluated how HCWs assess treatment adherence among their own patients, regarding their monitor and support strategies, as well as their general perception of treatment adherence in the Netherlands.

## METHODS

This was a quantitative web-based cross-sectional survey conducted from 1 December 2023 to 31 January 2024 in the Netherlands with an annual TB incidence of 4.1 per 100,000 population.^15^ Most reported TB cases (82%) in the country were in people of foreign origin.^15,16^

In the Netherlands, Municipal Public Health TB Services (MPHS) are responsible for implementation of TB control, focusing on active case finding through targeted screening of high-risk groups like migrants and asylum seekers, along with contact investigations and offering TPT to vulnerable groups like young children.^16,17^ Screening for TBI is performed in certain migrant groups from high-incidence TB countries. TB nurses provide treatment adherence support to individuals receiving community-based TB care.^16,18^ In 2022, 122 children and AYAs were diagnosed with TBD.^19^ A small percentage was referred to hospital to various specialities, such as (paediatric) infectious diseases, (paediatric) pulmonology, and paediatrics.

All HCWs in the Netherlands treating children and AYAs with TBD and TBI were eligible for participation. Professional societies with members treating this group agreed to distribute the survey link to their members: the MPHS, the Society for Physicians working in the TB departments (VvAwT), the Association of TB nurses (V&VN), the Dutch Society for Paediatricians (NVK), the Dutch Society for Physicians’ Infectious Diseases (NVII), and the TB coordinators of the Dutch Society for Pulmonary Physicians (NVALT).

Based on previous literature on adherence interventions, a pilot-tested questionnaire was developed as an electronic Case Report Form (eCRF) using the secure REDCap platform.^13,20^ The questionnaire contained data on HCWs’ baseline characteristics and methods for monitoring and supporting treatment adherence. Geographical data from participants were not requested as this could have easily led to the identification of the respondents. As this was an observational descriptive study, no formal power calculations were performed. Descriptive statistical analyses were conducted using IBM SPSS Statistics 28^®^.

The Medical Ethics Review Board of the University Medical Center Groningen (METc number UMCG, METc 2023/523) approved this study as not being clinical research with human subjects (Medical Research Involving Human Subjects Act [WMO]). Participants provided informed consent after receiving written study information. Data from the eCRF were anonymised using unique research identification numbers and contained no identifiable information.

## RESULTS

Ninety-three HCWs participated, with the majority from the MPHS (54%) and most being doctors (69%). Further characteristics of the participants are described in Table 1.

**Table 1.** Characteristics of study participants (n = 93).

Variables	Values	N (%)	Median (range)
Gender	Women	65 (69.9)	
Age (years)			51 (23–63)
Sector (more options possible)	University hospital	23 (24.7)	
	General hospital	23 (24.7)	
	Public health service (MPHS)	50 (53.8)	
Profession			
Public health service	TB control physician (n = 28)	18 (19.4)	
	TB control nurse (n = 65)	28 (30.1)	
Hospital care			
Adult care (≥18 years)	Pulmonologist (TB coordinator) (n = 77)	21 (22.5)	
	ID specialist (n = 114)	4 (4.3)	
Paediatric care (<18 years)	Pulmonologist (n = 2)	1 (1.1)	
	ID specialist (n = 86)	14 (15.1)	
	General paediatrician (n = 1221)	3 (3.2)	
Other (3 residents, 1 nurse)		4 (4.3)	
Experience in TB (years)			10 (0–35)
Type of TB monitored/supported by participant	TBD	9 (9.7)	
	TBI	1 (1.1)	
	Both	83 (89.2)	
Number of persons with TB disease and on TPT/year	TBD		10 (1–100)
	TPT		18.5 (1–200)
Age group of persons with TBD/TBI seen in daily practice	0–11 years	64 (68.8)	
	12–18 years	70 (75.3)	
	19–24 years	68 (73.1)	

ID = infectious disease; TBD = TB disease; TBI = TB infection; TPT = TB preventive therapy.

**Figure 1.**
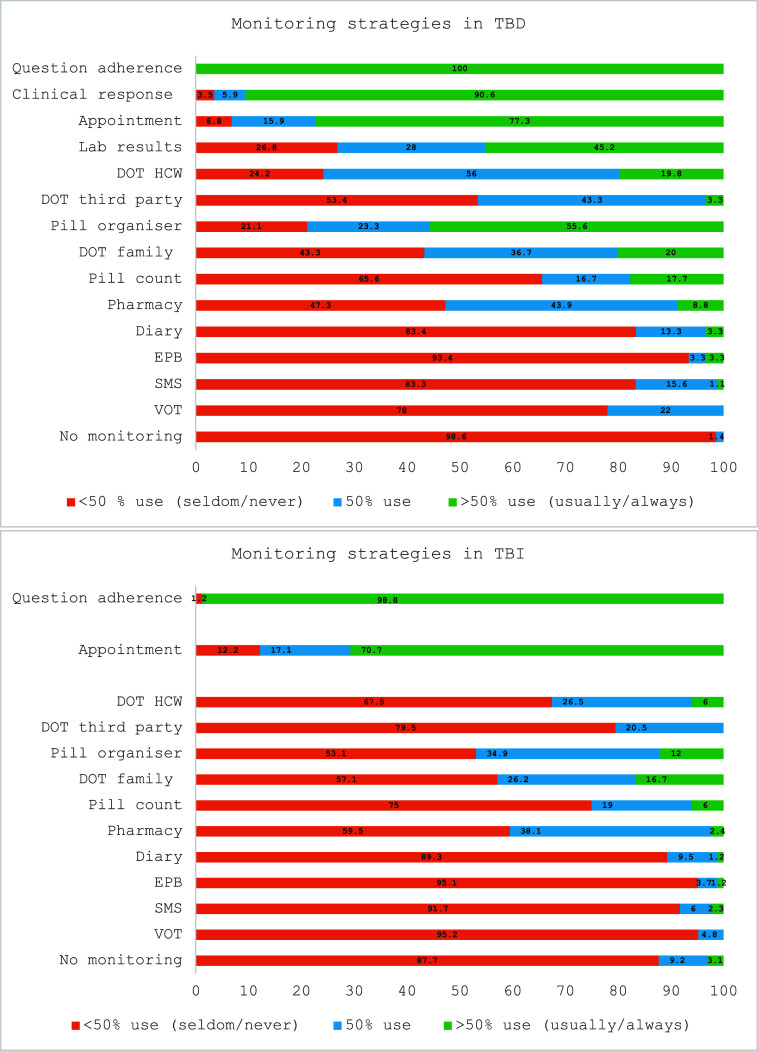
HCWs monitoring strategies for treatment adherence in TBD and TBI. DOT = directly observed therapy; EPB = electronic pill bottle; HCWs = health care workers; lab results = laboratory results; question adherence = question on adherence; SMS = SMS and WhatsApp; TBD = TB disease; TBI = TB infection; VOT = video-observed therapy.

The monitoring strategies reported to be used by HCWs for treatment adherence for TBD and TBI are shown in Figure 1.

For TB treatment, the most used strategies included verbal questioning on adherence (100%) and evaluating clinical responses (91%) during in-person consultations (77%). All HCWs employed an additional strategy to monitor treatment adherence, with the pill organiser (i.e., week box) being favoured by 56% of the participants. DOT by an HCW was usually or always used by nearly 20% of the participants, while 56% used it half the time. DTs were seldom or never used by 78% of the participants. VOT and SMS were used half the time by 22% and 16% of the participants, respectively.

For TPT, the most used strategy was also verbal questioning on adherence (99%) during in-person consultations (71%). Over 50% of the participants seldom or never used alternative strategies to monitor treatment adherence. If an additional strategy was employed, DOT by a family member or the use of a pill organiser was the most preferred option. DTs were seldom or never used by more than 90% of the participants.

**Figure 2.**
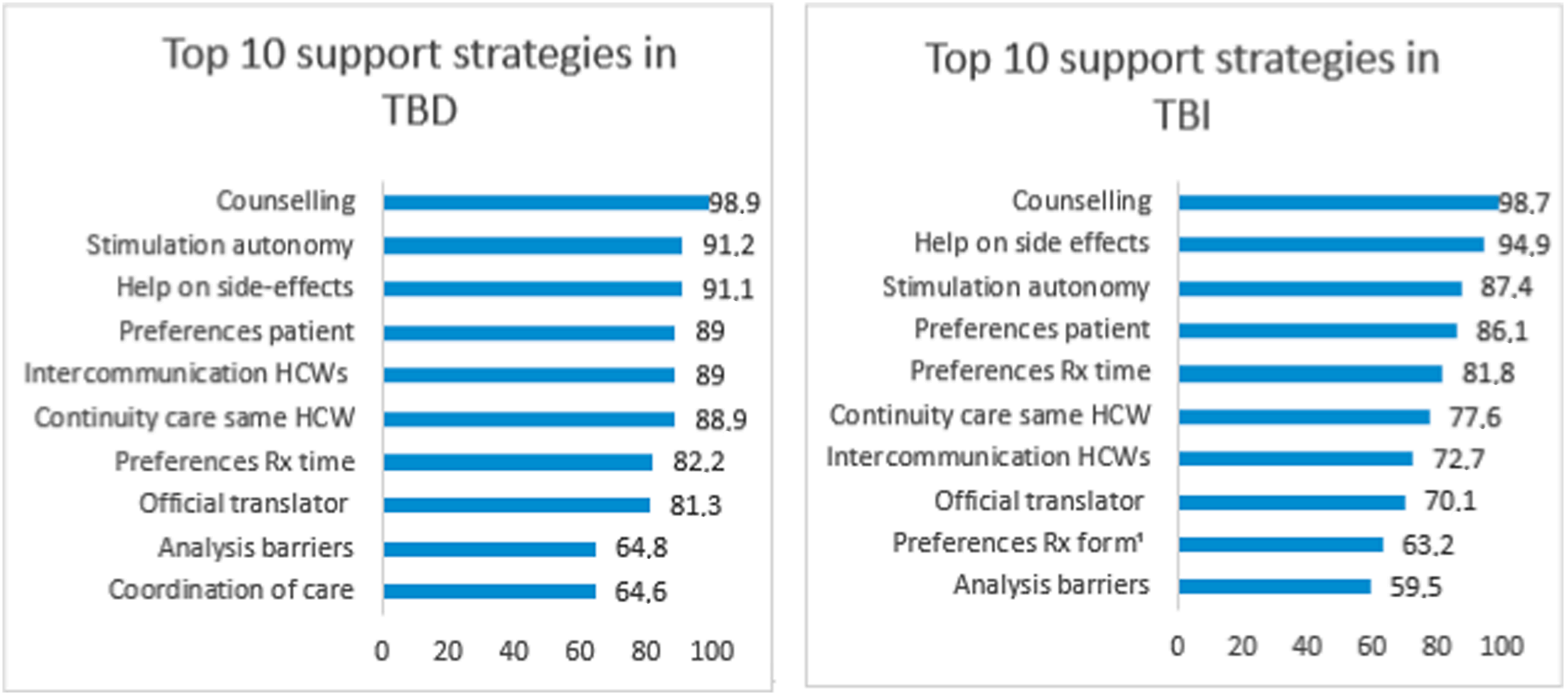
Top 10 support strategies in TBD and TBI used by participants in percentage. HCWs = health care workers; Rx = medication; TBD = TB disease; TBI = TB infection. ¹If applicable.

The various strategies to support treatment adherence for TBD and TBI are presented in Table 2. The top 10 reported strategies are shown in Figure 2, with counselling, stimulation of autonomy, and management of side effects being the most important. Financial support was the least used strategy. Additional data on support strategies are displayed in the Supplementary Data.

**Table 2.** Support strategies by HCWs to improve adherence to TPT and TB treatment.

Education/information	Counselling	Folders/website
Autonomy	Preferences of individual with TB	Preferences medication (Rx) time
	Stimulation autonomy	Preferences medication (Rx) form**^A^**
Cultural support	Use official translator	Use translation apps
Psychosocial support	Manage side effects	Analysis barriers adherence
	Identify/help psychological problems	Motivational interview
	Peer support	
Financial support	Use incentives	Use enablers
Organisation health care	Contact details of HCWs	Choice on time appointment
	Continuity of care with the same HCW	Decentralisation of care
	Coordination of care (i.e., appointments on the same day)	Support social interruption
	Short-waiting-time clinic**^A^**	Stigma reduction
	Intercommunication HCWs	Contact by SMS/WhatsApp

HCWs = health care workers; TPT = TB preventive treatment.

A If applicable.

**Figure 3.**
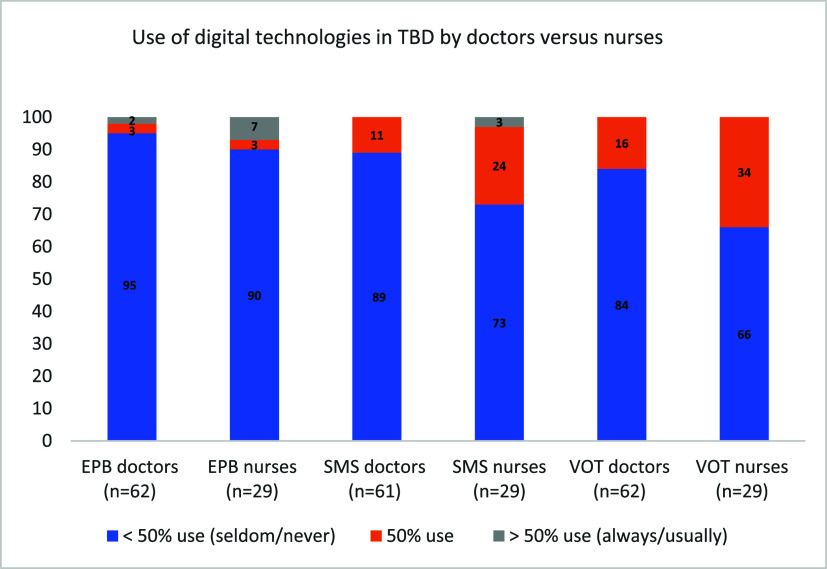
Use of DTs in monitoring TB treatment by doctors and nurses. DTs = digital technologies; EPB = electronic pill bottle; n = numbers; SMS = SMS or WhatsApp; TBD = TB disease; VOT = video-observed therapy.

TB nurses were reported to be the primary HCWs involved in monitoring and supporting adherence to TB treatment (79% of the participants) and TPT (75% of the participants). Nurses used additional monitoring strategies more often than doctors in the case of TB treatment, including pill organisers, prescription verification with pharmacies, pill counts, and DOT by themselves or third parties, as detailed in the Supplementary Data Table S3. Although nurses utilise DTs, like VOT and SMS, slightly more frequently than doctors, most of the nurses (66% or more across the various strategies) seldom or never utilise DTs, as shown in Figure 3.

The most frequently mentioned factors associated with low treatment adherence, leading HCWs to opt for supervised medication administration (like DOT by HCWs), included low health literacy (93%), challenging social circumstances (85% – e.g., homelessness), substance abuse (80%), psychological disorders (74%), and language barriers (65%). Conversely, the most frequently mentioned factors associated with good treatment adherence without the need for intensive monitoring include individuals with normal or good health literacy (90%), strong family/friends support (72%), and higher education levels (60%).

HCWs assessed an estimated adherence rate of 85% (range 58%–98%) among the persons with TB whom they were supporting, while they perceived a general adherence rate of 79% (range 49%–98%) for all persons with TB in the Netherlands. For TPT, they assessed an estimated adherence rate of 84% (range 49%–100%) in their own practice and 75% for TBI (range 36%–95%) in general in the Netherlands.

## DISCUSSION

This study assessed HCWs’ strategies for monitoring and supporting adherence in TB treatment and TPT among children and AYAs in a low-incidence country.

The most frequently mentioned method to assess treatment adherence in TBD was verbal questioning on adherence during in-person consultations and evaluation of clinical responses, always in combination with another monitoring strategy. The pill organiser was favoured by most participants, and approximately 20% of the participants usually or always used DOT by an HCW or family member.

Consistent with our study, a meta-analysis on interventions to improve treatment adherence in paediatric TB found that verbal questioning on adherence and clinical visits are common practises for assessing TB treatment adherence in children and adolescents, often combined with another adherence intervention strategy.^20^ However, the validity of self-reports can be questioned due to their susceptibility to social desirability and memory biases, which often lead to an overestimation of how consistently patients follow medication regimens.^21^ The reliability of self-report may be influenced positively if patient-provider communication is open-ended and non-judgemental.^22^

Next to self-reporting, pill organisers were often used to monitor treatment adherence. Pill organisers were mentioned as a potential best practice for improving adherence in TB in low-incidence European countries.^23^ However, the effectiveness of a pill organiser on treatment adherence has not been evaluated in children and AYAs with TB.

DOT by an HCW is usually or always used by nearly 20% of the participants to monitor treatment adherence in TBD. HCWs reported using DOT when factors such as low health literacy, challenging social circumstances, and language barriers are anticipated. The prevalence of TB among foreign-born persons, such as asylum seekers, likely explains the preference for DOT. However, the DOT strategy has been criticised for being paternalistic, costly, time-consuming, and subject to debate regarding its effectiveness.^24–26^

DTs were seldom or never used by the majority of the HCWs in this study. Although nurses used DTs more frequently than doctors, we had expected the majority of nurses to use them more often, given the widespread Internet access in the Netherlands and the potential effectiveness of these tools.^26,27^ A systematic review of eHealth in TB management found higher treatment completion rates and greater satisfaction among eHealth users compared with standard care, without increased costs. eHealth saved time by reducing travel and consultation durations compared with DOT.^27^ Another meta-analysis on adherence interventions in TB treatment suggested that electronic medication monitors could reduce LTFU and improve TB treatment outcomes.^13^ Since the WHO recommendations for using DTs to support TB, some high-incidence countries have started implementing or piloting these tools.^13,14,28,29^ The reasons for the limited use of DTs are unclear but interesting to explore in future studies. Potential barriers identified in the literature include concerns about confidentiality breaches, inadequate staff training, and scepticism about the effectiveness of digital adherence support.^27,30,31^ Additionally, uncertainty about when and for whom to use DTs alongside other strategies may hinder implementation.^31^

Digital adherence support should complement interactions between persons with TB and HCWs, not replace them, as part of a broader TB adherence strategy. Support should be tailored to each person’s (age-specific) needs, reflecting person-centred care.^30^

Our study shows that the same monitoring strategies are favoured for both TB treatment and TPT (Figure 1). Additional monitoring strategies are seldom or never employed for TBI by most of the participants. This finding aligns with previous survey results on TBI support practices in European low-incidence TB countries.^23^ The emphasis on monitoring TB treatment is understandable given that non-adherence increases the risk of poor outcomes, including risk of relapse, acquiring drug-resistant TB, and ongoing transmission of infection.^13^ However, providing TPT to children and adolescents is important as they are at higher risk of developing TBD after acquiring infection.^2–4^ Additionally, TPT is considered to be important for TB elimination, but adherence is challenging due to barriers like treatment duration, side effects, and being asymptomatic. Support could improve therapy completion.^5,32^ Our study did not explore why HCWs were not using additional strategies to assess TPT adherence.

This study reveals that over 86% of the participants use counselling on disease, stimulate autonomy, and manage side effects as support strategies in TBD and TBI (Figure 2). Education and counselling on disease are linked with higher adherence rates, which is endorsed by various national guidelines, and commonly practiced in TB care.^12,13,23^ Stimulating autonomy of the person with TB is crucial within a person-centred approach, as advocated by the WHO.^5^

This study has limitations. We had to design a bespoke questionnaire due to the lack of validated tools for assessing adherence interventions in children, AYAs, and adults. This may result in respondent misinterpretations, non-representative findings, or reporting bias. Secondly, the responses may reflect social desirability bias, with participants potentially overreporting favourable activities and underreporting less favourable ones.^33^ Thirdly, adherence strategies by age and their impact on adherence were not studied and are valuable topics for future research. The overall response rate could not be estimated and the overall group size was rather small, making it unclear how representative the respondents were of the target population. However the response rate from the MPHS was determined with reasonable accuracy. As the MPHS are primarily involved in TB care, a response rate of 28 out of 65 TB nurses (43%) and 18 out of 28 TB doctors (64%) was considered representative. Furthermore, although the response was anonymous, based on personal encounters, we believe the response rate was high among the small group of paediatric and adult specialists who routinely treat persons with TB.

A strength of the study lies in its focus on children and AYAs, as the health needs of this group have been historically neglected in TB care and research.^8^ To our knowledge, no studies have evaluated treatment adherence strategies in TB care specifically for children and AYAs, at least not in low-incidence countries. This study is a first step towards understanding how TB care is organised for children and AYAs with the aim of tailoring care to better meet the needs of this group and improve outcomes. Further research is needed to assess how children and AYAs with TB experience treatment adherence monitoring, including their digital support needs. Additional studies are needed to identify barriers to DT implementation and guide tailored support strategies.

In conclusion, this study provides insights into current strategies for monitoring and supporting treatment adherence in children and AYAs in a low-incidence TB country. It underlines the ongoing use of traditional methods for monitoring and supporting treatment adherence in TBD and TBI, although their effectiveness remains unknown. It shows that DTs are still only limitedly integrated into daily practice, despite their proven efficacy.
